# Differences in N-glycosylation of recombinant human coagulation factor VII derived from BHK, CHO, and HEK293 cells

**DOI:** 10.1186/s12896-015-0205-1

**Published:** 2015-09-18

**Authors:** Ernst Böhm, Birgit K. Seyfried, Michael Dockal, Michael Graninger, Meinhard Hasslacher, Marianne Neurath, Christian Konetschny, Peter Matthiessen, Artur Mitterer, Friedrich Scheiflinger

**Affiliations:** Baxalta Innovations GmbH, Uferstraße 15, A-2304 Orth/Donau, Austria; BaxaltaInnovations GmbH, Industriestraße 72, A-1220 Vienna, Austria; Baxalta Innovations GmbH, Donau-City Straße 7, 1220 Vienna, Austria

## Abstract

**Background & Methods:**

Recombinant factor VII (rFVII), the precursor molecule for recombinant activated FVII (rFVIIa), is, due to its need for complex post translational modifications, produced in mammalian cells. To evaluate the suitability of a human cell line in order to produce rFVII with post-translational modifications as close as possible to pdFVII, we compared the biochemical properties of rFVII synthesized in human embryonic kidney-derived (HEK)293 cells (HEK293rFVII) with those of rFVII expressed in Chinese hamster ovary (CHO, CHOrFVII) and baby hamster kidney (BHK, BHKrFVII) cells, and also with those of plasma derived FVII (pdFVII), using various analytical methods. rFVII was purified from selected production clones derived from BHK, CHO, and HEK293 cells after stable transfection, and rFVII isolates were analyzed for protein activity, impurities and post-translational modifications.

**Results & Discussion:**

The analytical results showed no apparent gross differences between the various FVII proteins, except in their N-linked glycosylation pattern. Most N-glycans found on rFVII produced in HEK293 cells were not detected on rFVII from CHO and BHK cells, or, somewhat unexpectedly, on pdFVII; all other protein features were similar. HEK293rFVII glycans were mainly characterized by a higher structural variety and a lower degree of terminal sialylation, and a high amount of terminal N-acetyl galactosamines (GalNAc). All HEK293rFVII oligosaccharides contained one or more fucoses (Fuc), as well as hybrid and high mannose (Man) structures.

**Conclusions:**

From all rFVII isolates investigated, CHOrFVII contained the highest degree of sialylation and no terminal GalNAc, and CHO cells were therefore assumed to be the best option for the production of rFVII.

**Electronic supplementary material:**

The online version of this article (doi:10.1186/s12896-015-0205-1) contains supplementary material, which is available to authorized users.

## Background

Patients with hemophilia A and B who have developed inhibitory antibodies against factor VIII (FVIII) or factor IX (FIX), are successfully treated with by-passing agents such as FEIBA, an activated prothrombin complex concentrate derived from human plasma [1], or recombinant activated factor VII (rFVIIa), to control and prevent bleeding. FVII [UniProt: P08709] is a vitamin K dependent serine protease of approximately 55 kDa. In its zymogen form, FVII is a single chain protein which is cleaved once between R152 and I153 upon activation to its enzyme form FVIIa, consisting of a light and a heavy chain linked by one disulfide bond. FVII carries complex post-translational protein modifications (PTMs) including γ-carboxylation, N- and O-linked glycosylation, and β-hydroxylation [2]. Some of these modifications have been described as necessary for secretion of rFVII [3], interaction with tissue factor (TF) [4, 5], and platelet surface interactions between FVIIa/TF and its substrate factor X [6].

Due to its complex PTMs, rFVII is expressed in genetically engineered mammalian cell lines and activated to rFVIIa during purification. The modifications of rFVIIa produced in Chinese hamster ovary (CHO) and baby hamster kidney (BHK) cells have been described to differ from those of pdFVII or FVIIa, especially in N-glycosylation. N-glycans on pdFVII possess a higher amount of tri- and tetra-antennary structures and a higher degree of sialylation than CHOrFVIIa and BHKrFVIIa. BHKrFVIIa also has a lower degree of γ-carboxylation on the eleventh Gla residue than pdFVII [7], which does not influence biological activity [8]. Side–by–side assessment of pdFVIIa and BHKrFVIIa showed similar *in vitro* and *in vivo* efficacy in rabbits [9]. O-glycans on S52 and S60 were found to differ on pdFVII and BHKrFVII [10], and terminal N-acetyl galactosamines (GalNAc) were detected only on BHKrFVIIa [7, 11, 12]. *In vivo* studies directly comparing the pharmacokinetics of FVII and/or FVIIa forms with dissimilar glycosylation patterns without altering their protein sequence were conducted in rats [13, 14] and humans [15, 16], and uniformly showed differences between pdFVII, CHOrFVIIa, and BHKrFVIIa, and desialylated rFVIIa versus native BHKrFVIIa. In rats, desialylated BHKrFVIIa showed 26 % of the half-life of native BHKrFVIIa [13], and BHKrFVIIa had an *in vivo* recovery (IVR) that was 56 % of that of CHOrFVIIa [14]. In a pharmacokinetics study in healthy subjects and hemophilia patients, CHOrFVIIa showed a significantly higher activity level than BHKrFVII (for example, the area under the curve of CHOrFVIIa was 1.28-fold that of BHKrFVIIa in hemophilia patients) [16]. BHKrFVIIa showed a shorter half-life and a higher volume of distribution in FVII-deficient patients than pdFVII [15].

Several *in vitro* and *in vivo* studies demonstrated that receptors and inhibitors are involved in binding of rFVII and rFVIIa including the asialoglycoprotein receptor on liver cells [17–19], and that most rFVIIa is cleared in complex with its inhibitor antithrombin III when intravenously administered to hemophilia patients [20]. In mice, the elimination kinetics of FVII were not affected by activation and subsequent inactivation by inhibitor complex formation [17]. rFVIIa was shown to enter the extravascular compartment in mice after intravenous application [21], and to be internalized into human platelets and re-exposed again on their surface [22], both mechanisms which might explain its prolonged pharmacological effect beyond the circulatory half-life.

The present study evaluated the potential of using a human cell line to synthesize a rFVII with more human-like post-translational modifications than those derived from rodent cell lines. Here, we describe the modifications on rFVII expressed in human embryonic kidney-derived (HEK)293 cells compared with those of rFVII derived from CHO and BHK cell lines and of pdFVII.

## Methods

### Proteins

pdFVII was purchased from Haematologic Technologies Inc. (Essex Junction, VT, USA), commercially available rFVIIa (NovoSeven) from NovoNordisk (Bagsvaerd, Denmark).

### Expression plasmids

The construction of the plasmid vector pselp/huFVII encoding human FVII is described elsewhere [23]. This vector was used as a template to generate a PCR product including a Kozak sequence for insertion via restriction sites into a standard mammalian expression plasmid vector containing the hygromycin resistance gene. The resulting vector pFVIIhyg was used for mammalian cell line transfection. A murine dihydrofolate reductase (DHFR) cDNA was cloned from the original plasmid containing the full-length DHFR cDNA termed pAdD26SV(A)-3 [24] into an in-house plasmid (derivative of pSV40β, Clontech, Mountain View, CA, USA). The resulting plasmid pSVDHFR was used as a selection plasmid for DHFR-deficient CHO cells.

### Cell culture

BHK (BHK-21; ATCC#CCL-10™) and HEK293 cells (ATCC#1531) were provided by the American Type Culture Collection (Manassas, VA, USA), CHO DXB11 by Columbia University, New York. All cells were cultivated in DMEM/Ham’s F12 medium (Life Technologies, Carlsbad, CA, USA) containing 2–10 % fetal bovine serum (FBS, PAA, Linz, Austria). BHK and HEK293 cells were transfected with the plasmid vector pFVIIhyg using calcium phosphate co-precipitation [25]. Producer clones were selected by antibiotic resistance and limited dilution cloning. CHO DXB11 cells were co-transfected with human FVII- and murine dihydrofolate-reductase-encoding plasmids pFVIIHyg and pSVDHFR by calcium phosphate co-precipitation, and cultivated in DMEM/Ham’s F12 lacking the purine and pyrimidine precursors hypoxanthine, glycine, and thymidine, and containing dialyzed FBS (PAA). Producer clones were generated by methotrexate (MTX, Ebewe, Unterach, Austria) gene co-amplification [26] followed by limited dilution cloning, and selected according to cell-specific productivities and high antigen and activity titers at low pre-activation, and to band pattern in SDS-PAGE. rFVII was produced from cell lines growing adherently in roller bottles or triple-T-flasks (both from Nunc, Thermo Fisher Scientific, Waltham, MA, USA) after withdrawing FBS, and, after washing cells once with phosphate-buffered saline without Ca and Mg (Life Technologies), in medium containing 10 μg/mL vitamin K1 (Sigma, St. Louis, MO, USA) for 24 h. Harvests were taken every 24 h for up to 1 week.

### Purifications

Filtered cell culture supernatants containing rFVII were purified using multiple sequential ion-exchange chromatography steps.

### Protein activity and concentration

FVII activity was measured by prothrombin time (PT) clotting assay using FVII-deficient plasma and STA Neoplastin Plus (both from Roche, Basel, Switzerland) as activator. The FVII reference and control standards, and imidazole dilution buffer containing 1 % human serum albumin were from Baxter (Vienna, Austria). The reference and control standards had been calibrated against the 1^st^ or 2^nd^ International Standard for FVII concentrate. FVII pre-activation in culture supernatants was measured using a soluble truncated rTF as FVIIa-selective cofactor [27] by STACLOT FVIIa-rTF-kit (Diagnostica Stago, Asnieres, France), including FVII deficient plasma, recombinant soluble TF-phospholipids, and a FVIIa calibrator and controls. All clotting assays were performed on a STA compact automated coagulometer (Diagnostica Stago). FVII-antigen was measured by enzyme-linked immunoassay (ELISA) in 96-microwell plates (Thermo Scientific, Waltham, MA, USA) using sheep polyclonal antibodies (Enzyme Research Laboratories, South Bend, IN, USA, and Cedarlane, Burlington, ON, Canada) against human FVII for capture and detection, the latter conjugated to horse-radish peroxidase. pdFVII was used as a standard to establish a calibration curve.

### SDS-PAGE and Western blotting

Impurity screening of cell culture supernatants from selected clones and of the purified FVII preparations was carried out using SDS-PAGE with Western blotting. If not otherwise stated, chemicals were from Sigma. Molecular mass standards were from BioRad (Hercules, CA, USA). For Western blotting of cell culture supernatants, 50 ng FVII-antigen per lane from each FVII(a) sample were run on a 12 % bis/tris SDS-PAGel (NuPage, Life Technologies) at reducing conditions using dithiothreitol (DTT). The proteins contained in the gels were then blotted onto a nitrocellulose membrane (GE Healthcare, Uppsala, Sweden) for staining with a sheep anti-human FVII polyclonal affinity-purified antiserum (Affinity Biologicals, Ancaster, ON, Canada) and with an alkaline phosphatase-labeled rabbit anti-sheep IgG (H+L) affinitiy-purified secondary antiserum (Jackson Immuno Research, West Grove, PA, USA). Membranes were developed using BCIP/NBT. For impurity detection by silver staining, 220 ng per lane purified rFVII(a) was run on an 8 – 18 % gradient tris/acetate SDS-PAGel (ExcelGel kit, GEHealthcare) at reducing conditions and stained.

### Monosaccharide analysis

Monosaccharide analysis of FVII was performed with and without PNGaseF digestion to differentiate between N- and O-glycan originating components. All samples were desalted by acetone precipitation. For O-glycan monosaccharide composition, samples were digested with PNGase F (Prozyme, Hayward, CA, USA) at 37 °C overnight. Liberated N-glycans were removed by ethanol precipitation. All samples were subjected to acidic hydrolysis using 2 M trifluor-acetic acid (TFA, Applied Biosystems, now Thermo Fischer Scientific, Waltham, MA, USA) at 100 °C for 6 h and subsequently vacuum dried. Separation was carried out by ion exchange HPLC on a CarboPac PA20 column [28] using 4 mM sodium hydroxide under isocratic conditions with pulsed amperometric detection (HPAEC-PAD, Dionex, Sunnyvale, CA, USA).

### Oligosaccharide profiling

Oligosaccharides resembling sialylated and asialo N-glycans were separated after enzymatic liberation from the peptide backbone by high pH anion exchange chromatography and recorded using pulse amperometric detection (HPAEC-PAD) similarly as in [29]. The FVII preparations were desalted using acetone precipitation. After lyophilization of the protein pellet the samples were reconstituted in 50 mM sodium phosphate buffer pH 7.5 and incubated overnight with 6 mU PNGase F (Sigma) at 37 °C. Liberated N-glycans were isolated using ethanol precipitation. N-glycans were reconstituted in a water/acetic acid mixture and incubated at room temperature for 2 h. The samples were then vacuum dried and dissolved in water before measurement. Separation was performed by ion exchange HPLC using a CarboPac PA100 column (Dionex) with a sodium acetate/sodium hydroxide gradient with pulsed amperometric detection (Dionex).

### Sequence and post-translational modification analysis by LC-MS

Peptide maps of FVII-isolates were generated by chromatographic separation of tryptic peptides, and subsequent mass spectrometric analysis enabled relative quantification of γ-carboxylation, β-hydroxylation, and of N-linked and O-linked glycans. Tryptic digest was carried out using 50 μg of each FVII preparation. Samples were reduced by DTT (Sigma) and alkylated by iodoacetic acid (Sigma) prior to digestion. Tryptic digestion was carried out in 50 mM ammonium-bicarbonate buffer pH 8.4 (ammonium bicarbonate from Fluka) by addition of 2.5 μg trypsin (Roche) at 37 °C for 18 h. The reaction was stopped by addition of phosphoric acid. Tryptic peptides were separated by reversed phase HPLC (Agilent, Santa Clara, CA, USA) using a Jupiter C18 column (Phenomenex, Torrance, CA, USA) with an acetonitrile/water/TFA gradient coupled on-line to an electrospray-QTOF-MS (QTOFmicro, Waters, Milford, MA, USA). Detection was carried out using a diode array detector (Agilent) at 214 nm (scan width from 190 to 600 nm). All relative quantifications of the respective non-modified and modified peptides were performed in the positive ion mode with an acceleration voltage of 30 V and scanning range of 300 – 2,000 Da, and were analyzed with MassLynx 4.0 software (Waters). Internal calibration was carried out via ammonium phosphate clusters. Theoretical masses of post-translationally modified peptides were calculated manually using Excel 2007 software (Microsoft, Redmond, WA, USA). N-glycan structures were drawn using GlycoWorkBench software [30].

## Results

### Expression of rFVII in mammalian and human cell lines

**Fig. 1:**
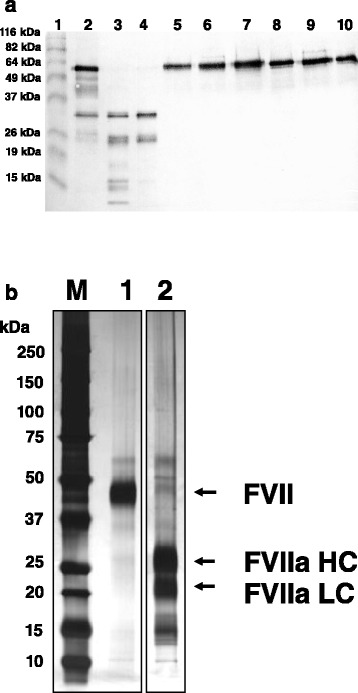
SDS-PAGE analysis of rFVII in cell culture supernatants and after purification. **a** Western blot after SDS-PAGE stained for human FVII: lane 1: molecular mass marker. pdFVII (lane 2), pdFVIIa (lane 3), commercially available rFVIIa (lane 4), supernatants from CHOrFVII clones (lanes 5–7) and from HEK293rFVII clones (lanes 8–10). 50 ng rFVII based on antigen were loaded per lane and resolved under reducing conditions by SDS-PAGE. Human FVII was detected by dual-antibody staining after blotting on a nitrocellulose membrane. Molecular mass marker sizes are shown on the left. **b** Silver-stained SDS-PAGE: 220 ng per lane each purified HEK293rFVII (lane 1), commercially available rFVIIa (lane 2), were resolved by SDS-PAGE and protein bands were made visible by silver staining. M: molecular mass standard, molecular mass marker sizes are shown on the left.. The FVII whole molecule, the heavy (HC) and light chain (LC) of rFVIIa are marked with arrows

Human rFVII derived from BHK, CHO, and HEK293 cells was produced at laboratory scale, and purified from culture supernatants. Producer cell lines were generated after several rounds of limited-dilution cloning, and derived from single cells. Specific clotting activities determined by PT clotting assay and antigen concentrations for rFVII preparations and pdFVII were ~2 kU/mg (Table 2). Cell-specific productivities of HEK293 and BHK cell clones were directly comparable using the same vector configuration and media, whereas CHO cell clone expression levels were augmented by gene co-amplification by stepwise concentration increases of the DHFR inhibitor MTX. The statistics of average rFVII productivities achieved by all three cell lines are summarized in Table 1 for a representative cell line development campaign. After three rounds of limited dilution cloning, BHK clones produced approximately one tenth of the average amount of rFVII per mL culture medium and day as produced by HEK293 and amplified CHO clones, which was similar. The level of pre-activation varied substantially within individual clones (ranges for clones from the third cloning round: CHO from 2 to 11 %, BHK from undetectable to 4 %, HEK293 from undetectable to 85 %). Appropriate clones for production of rFVII had therefore to be carefully selected for this parameter. The structural integrity of CHOrFVII and HEK293rFVII secreted into supernatants by selected clones was assessed by immunoblotting after SDS-PAGE; bands representing free light or heavy chains in reducing gels indicated no remarkable differences in fragmentation or pre-activation between selected clones from both cell types (Fig. 1a).

**Table 1:** Productivities of rFVII from BHK-, CHO- and HEK293-clones after two and three cloning rounds

Cell line	Number of limited dilution cloning round	Number of clones in first screening	Number of clones tested for final characterization	rFVII titer [IU/ml]	rFVII cell-specific productivity [IU/10^6^cells/day]	Pre-activation: IU rFVIIa per IU rFVII [%]
BHK	2	96	6	0.7 ± 0.2	0.5 ± 0.1	2.5 ± 0.5
	3	150	22	0.6 ± 0.3	1.1 ± 0.4	1.2 ± 0.9
CHO	2	143	7	1.0 ± 0.8	1.7 ± 1.8	8.3 ± 1.9
	3	198	46	5.7 ± 3.5	6.7 ± 3.1	5.6 ± 2.6
HEK293	2	235	36	5.8 ± 3.0	4.7 ± 1,6	4.2 ± 2.9
	3	132	35	6.7 ± 3.7	4.1 ± 2.4	11.6 ± 19.3

Mean values and standard deviations are shown for finally characterized clones obtained by limited dilution cloning. rFVII secreted into cell-culture supernatants was determined using a PT clotting assay, and the amount of pre-activated rFVIIa by STA-clot FVIIa-rTF assay. Supernatants were harvested after 24-h secretion into medium not containing FBS (see Methods)

rFVII was purified from cell culture supernatants in a multiple-step chromatographic procedure using ion-exchange resins at step yields of >50 %, resulting in 95 % pure rFVII as estimated from SDS-PAGE analysis applying silver staining or anti FVII Western blotting. SDS-PAGE analysis of purified HEK293rFVII compared with commercially available rFVIIa using silver staining is provided in Fig. 1b.

### Protein characterization

**Fig. 2:**
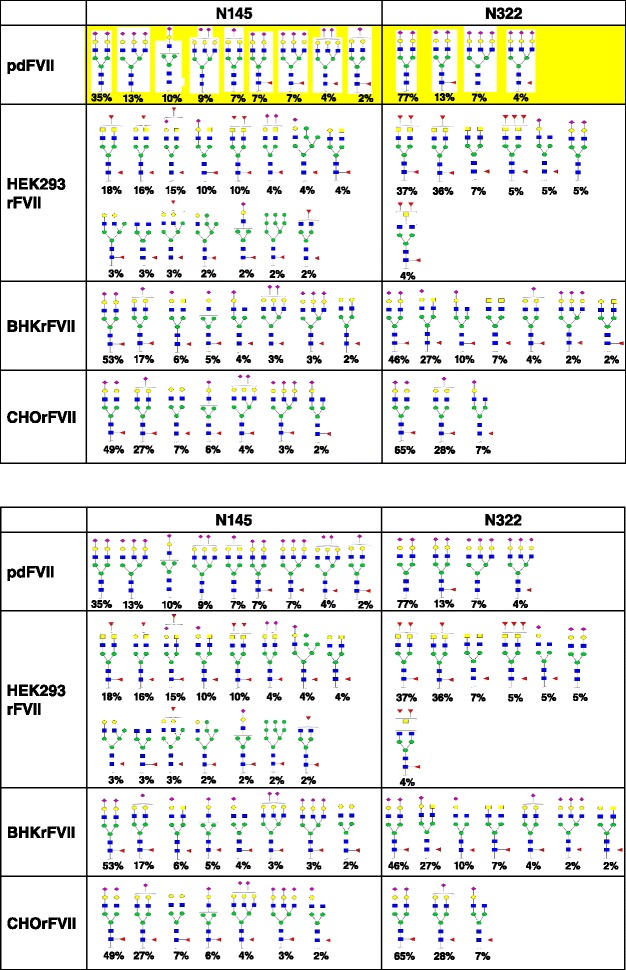
Proposed N-glycan structures on N145 and N322 for pdFVII, HEK293rFVII, BHKrFVII, and CHOrFVII. Legend: GlcNAc, Man, Gal, Fuc, GalNAc, sialic acid (NANA). Relative abundances of N-glycan structures at each N-glycosylation site detected by LC-MS are shown. Mean values four clones of HEK293rFVII, two clones of CHOrFVII, and values for one BHKrFVII clone and one pdFVII preparation are shown. Only structures detected at equal or higher 2 % are shown. The data for CHOrFVII, HEK293rFVII and BHKrFVII of each clone and of pdFVII can be found in Additional file 1: Table S1 in the supplementary file

**Fig. 3:**
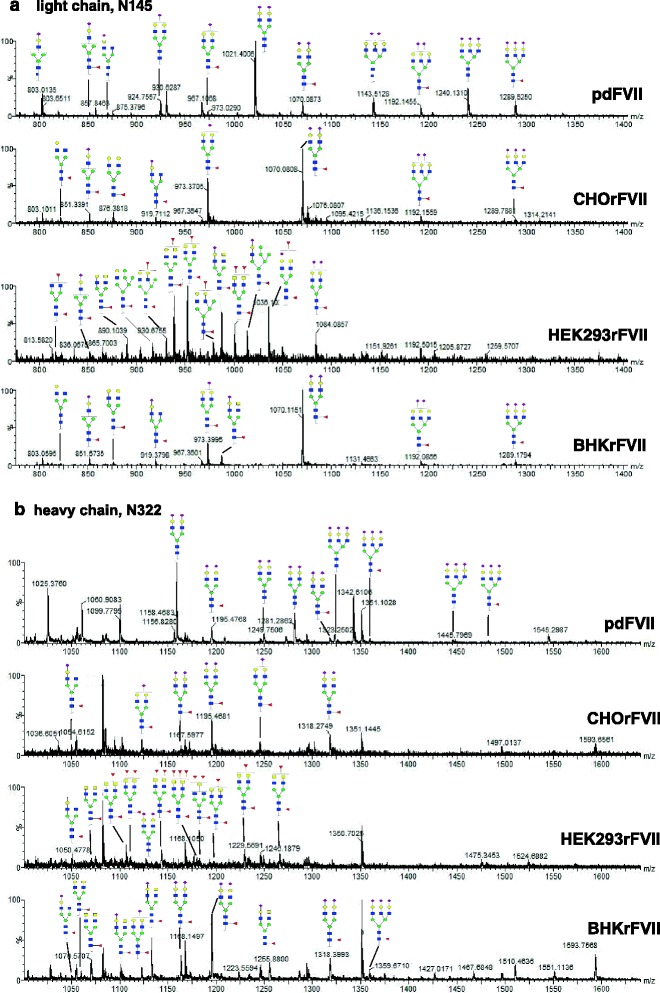
**a** and **b** Mass spectra of the N-glycosylated peptides containing the light and heavy chain glycosylation sites (N145 and N322) of pdFVII and rFVII isolated from CHO, HEK293 and BHK. **a** Mass spectra of the light chain peptides (N145). Relative intensities of glycan structures were calculated based on the masses of the 3-fold positively charged peptides. **b** Mass spectra of the heavy chain peptides (N322) based on the 4-fold positively charged peptides. Both mass spectra of all samples were recorded within the same system setup. Only intensities of manually identified peaks were used for the calculation of relative amounts of N-glycan species. Major identified oligosaccharide structures are shown for each FVII isolate. Theoretical and calculated molecular masses, and relative abundances of glycan structures can also be found in the Additional file 1: Tables S4A-H

**Fig. 4:**
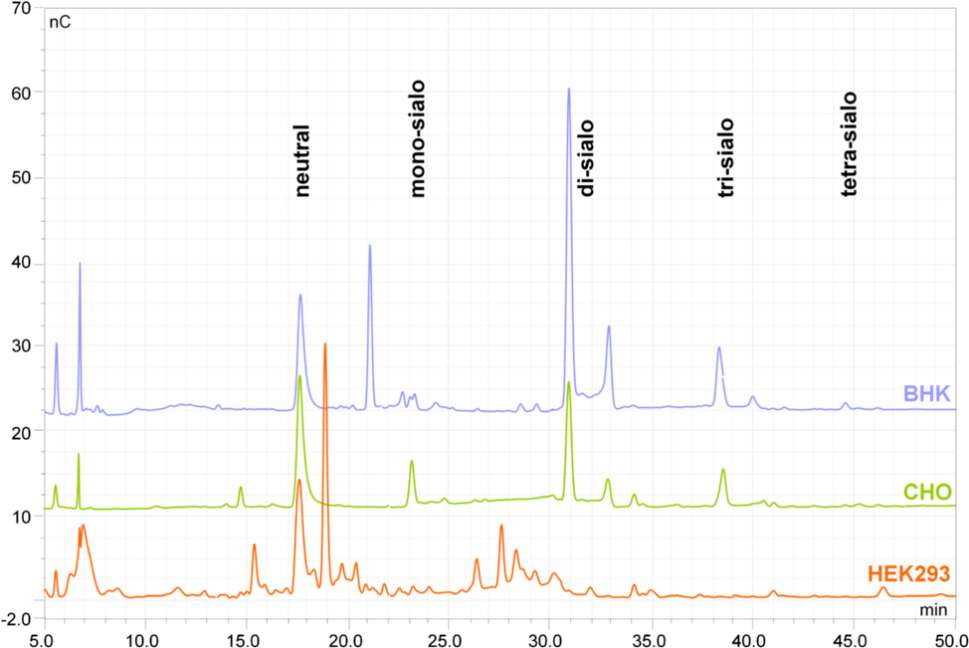
Oligosaccharide maps of BHK-, CHO-, and HEK293-derived rFVII. N-linked oligosaccharides obtained after PNGase F digest were separated according to their charge by HPAEC with PAD. Positions of neutral and bi-antennary/mono-sialylated to tetra-antennary/tetra-sialylated structures are marked

**Fig. 5:**
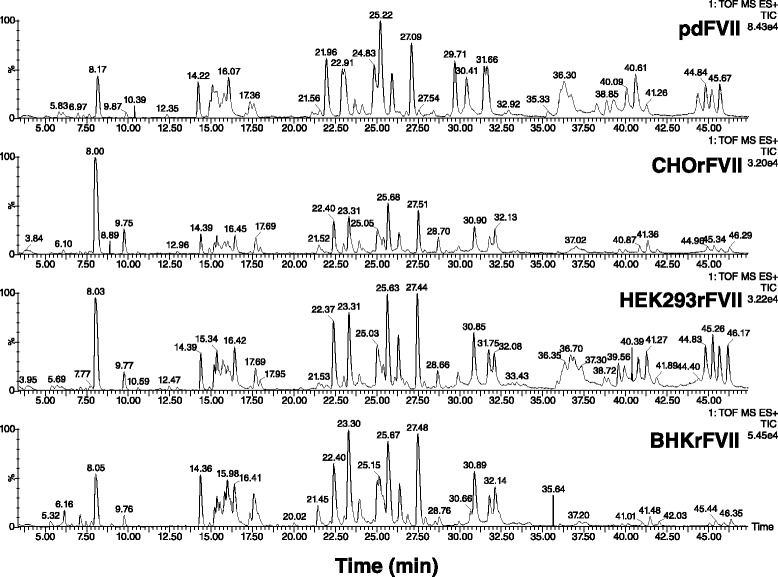
Peptide maps of CHO-, HEK293-, BHK-derived rFVII, and pdFVII. Peptides were separated by HPLC on a C18 column after reduction, alkylation and tryptic digest. Shown are chromatograms with retention times of respective peptides detected at 214 nm of one representative preparation of each FVII isolate, all four recorded within one sequence

**Fig. 6:**
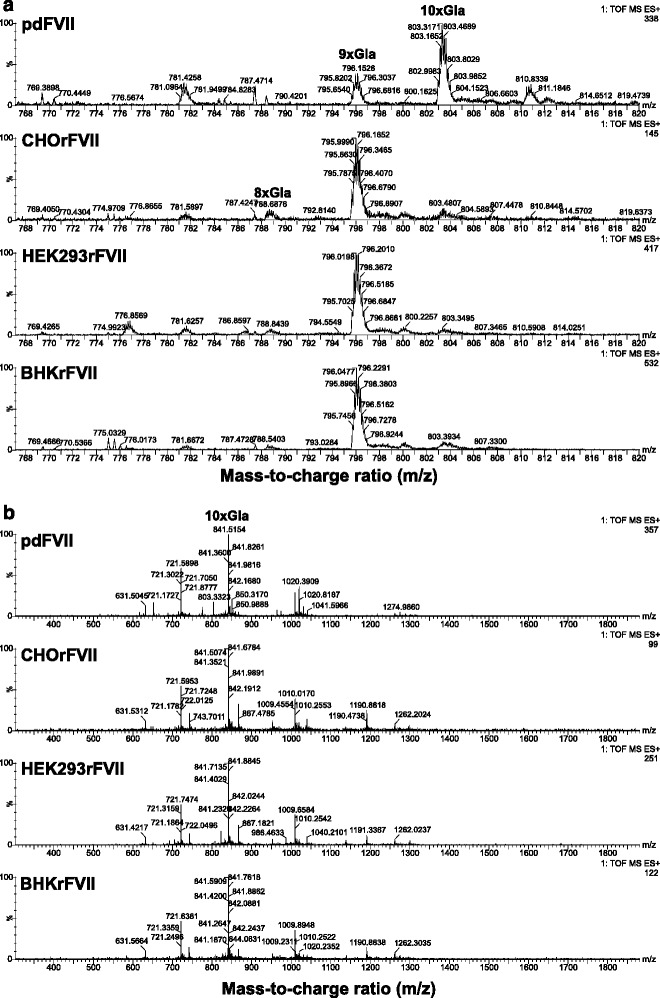
**a** and **b** Mass spectra of γ-carboxylated peptides of CHO-, HEK293-, BHK-derived rFVII, and pfVII. Shown are mass spectra of peptides T1-5 (**a**) with peaks corresponding to peptides containing 8, 9, and 10 Gla residues indicated, and T1-6 (**b**), which mostly contained 10 Gla residues. All spectra were recorded within one system setup. Corresponding data can also be found in Additional file 1: Tables S1A-D in the supplementary file

Supernatants of four clones from HEK293 and two clones from CHO and one from BHK were purified independently and analyzed for post-translational modifications. Mean values for all parameters are shown in Table 2, except for N-glycan analysis which is shown in Figs. 2, 3a and b and 4. Apart from N-glycosylation, identical protein structures and insignificantly different post-translational modifications were detected between rFVII from BHK, CHO, and HEK293 cells, and pdFVII. Tryptic peptide analysis resulted in highly similar peptide maps for all FVII-isolates, demonstrating primary amino acid sequence identity (Fig. 5). The absence of propeptide was confirmed for all isolates by LC-MS. Degrees of γ-carboxylation were assessed by LC-MS for the tryptic peptides representing the Gla domain which showed, in all cases, eight fully modified Gla residues. The ninth Gla was modified by 96 – 98 % in all FVII isolates, and the tenth Gla similarly modified by 50 % in all rFVII isolates (BHKrFVII: 53 % (*n* = 1 clone); CHOrFVII: 53 % (mean of 2 clones); HEK293rFVII: 56 ± 3.7 %; mean ± standard deviation of 4 clones), and by 82 % in pdFVII. A comparison of mass spectra of peptides T1-5 and T1-6 from all FVII isolate samples is given in Fig. 6a and b. Raw data from LC-MS are provided in the Additional file 1: Table S1A-D. Clonal variability is shown in Additional file 1: Table S2. The frequency of β-hydroxylation of D63 was similar within rFVII isolates but higher (~10 %) than for pdFVII (<1 %).

**Table 2:** Summary of protein characteristics and post-translational modifications of pdFVII and rFVII from three cell lines

	pdFVII	CHOrFVII	HEK293rFVII	BHKrFVII
rFVII specific activity calculated from PT clotting values per mg protein (antigen) [1000 IU/mg]	Not done, theoretical specific activity 2.0 kIU/mg	2.0	2.3	1.8
Peptide map	Similar			
γ-carboxylation of E35; i.e. abundance of 10^th^ Gla [%]	82 %	53 %	56 %	53 %
Presence of propeptide	Not detected by peptide mapping			
Hydroxylation of D68 [%]	<1 %	9 %	7 %	15 %
O-glycosylation on S52 / S60; only three major identified types shown [%]	31 %: Glc(Xyl)_2_ / Fuc	56 %: Glc(Xyl)_2_ / Fuc	62 %: Glc(Xyl)_2_ / Fuc	21 %: Glc(Xyl)_2_ / Fuc
	27 %: GlcXyl / Fuc	7 %: GlcXyl_2_	7 %: GlcXyl / Fuc	50 %: Glc / Fuc
	15 %: Glc / Fuc	14 %: Glc / Fuc	10 %: Glc / Fuc	12 %: Glc / -

Peptide mapping and the PTMs were done by LC-MS after tryptic digest (see Methods). The raw data for γ-carboxylation and O-glycosylation of all FVII isolates can be found in the addional PDF file provided in Additional file 1: Tables S1 and S3, respectively. Legend: *Fuc* Fucose, *Gla* γ-carboxy glutamic acid, *Glc* Glucose, *IU* International units, *Xyl* Xylose

**Fig. 7:**
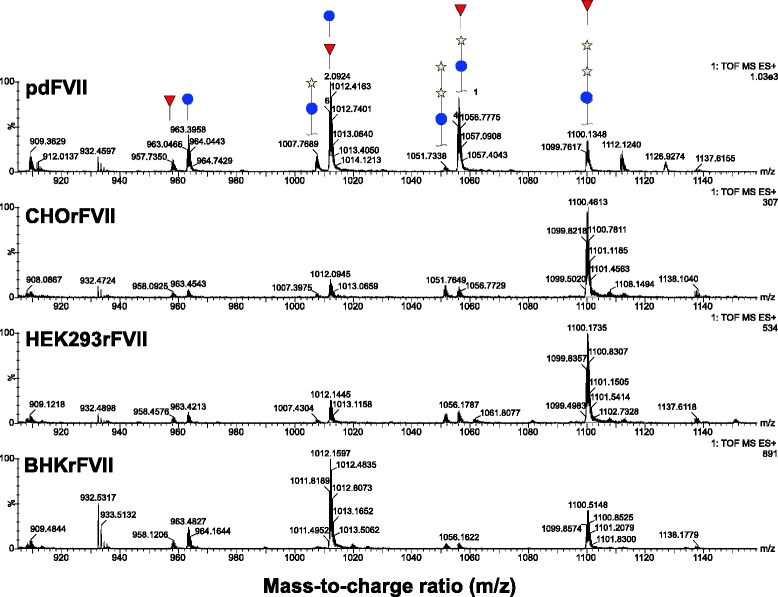
Mass spectra of the O-glycosylated peptide T7 detected from CHO-, HEK293-, BHK-derived rFVII, and pdFVII. Shown are masses of the T7 peptide corresponding to the peptides containing O-glycans on S52 and S60. The major forms on CHOrFVII and HEK293rFVII were GlcXyl2 on S52 and Fuc on S60, whereas BHKrFVII contained mostly one Glc on S52 and Fuc on S60, and pdFVII mostly GlcXyl or GlcXyl2 on S52 and Fuc on S60 and. Symbols: Fucose; Glucose; Xylose. In Additional file 1: Tables S3A-D, raw data can be found

The distribution of O-glycosylation structures analyzed at the peptide level by LC-MS was similar for all rFVII and pdFVII isolates except BHKrFVII which had a lower degree of xylose (Xyl). Figure 7 shows the mass spectra of the peptide T7 containing O-glycosylation sites S52 and S60 for all FVII isolates. Raw data from LC-MS for O-glycosylation are provided in Additional file 1: Tables S3A-D. rFVII with a single glucose (Glc) on S52 and a single fucose (Fuc) on S60 was the major form in BHKrFVII (50 %). pdFVII contained 60–70 % of Xyl-containing oligosaccharides on S52 similarly as described in [11], as did CHOrFVII and HEK293rFVII. The presence of Glc and Xyl was confirmed by monosaccharide analysis.

Oligosaccharide profiling by HPAEC-PAD revealed typically charged peak groups corresponding to 0 to 4 sialic acids on the N-glycans of CHO, BHK-derived rFVII, and HEK293rFVII (Fig. 3). CHOrFVII and BHKrFVII showed a similar N-glycan pattern, which was different to that for HEK293rFVII, whose main structures were composed of neutral to di-sialylated N-glycans at a higher diversity.

The two N-glycosylation sites, one each on the heavy (N322) and the light chain (N145), were characterized by LC-MS of relevant modified peptides after tryptic digestion. Sialic acid quantification was done by calculation from the LC-MS data.. The degree of sialylation on our pdFVII preparation was 4.06 sialic acids per molecule for pdFVII (1.93 on N145 and 2.13 on N322). These values were lower for the light chain and almost equal for the heavy chain as described in the literature (2.55 on N145 and 2.10 on N322, and in total 4.65, respectively) [11]. All rFVII preparations had a lower degree of terminal sialic acids than pdFVII: 3.14 ± 0.06 (mean ± standard deviation; *n* = 2) sialic acids per molecule for CHOrFVII (1.49 ± 0.06 plus 1.65 ± 0.01), 2.96 for BHKrFVII (1.57 and 1.39, one preparation only), and 0.57 ± 0.07 (*n* = 4) for HEK293rFVII (0.43 ± 0.04 and 0.14 ± 0.06).

Results for relative abundances of proposed structures for all FVII isolates are shown in Fig. 2; more detailed data are provided in Additional file 1: Table S4A-H; representative spectra for the peptides containing the light and heavy chain N-glycosylation sites are shown in Fig. 3a and b. HEK293rFVII, for which isolates from four clones were available, showed relative standard deviations of 4 to 56 % for each N-glycan structure. It should be noted that the variation was a sum of clonal variation, rFVII preparation, and analytical variation.

For pdFVII, reference values from the literature were compared with our own findings: The majority of N-glycans on pdFVII were complex-type bi- and tri-antennary without core-fucosylation. The main N-glycan structure on the light and heavy chain was identified as a complex-type biantennary disialylated N-glycan. Sialylation was almost complete and showed mono- to trisialylated structures. A higher diversity of structures on the light chain site, N145, was also found. This complexity on the light chain was also observed on all rFVII preparations, probably due to co-translational glycosylation of the N145 site, and post-translational glycosylation of the N322 site [31]. On the light chain site, we detected a lower amount of tri- and no tetraantennary structures in our pdFVII preparation in contrast to 1 % tetraantennary forms described in the literature [11]. On the heavy chain site, the types and amounts of N-glycans detected were almost identical to literature values.

The main N-glycan structure for the BHKrFVII preparation on both the light (53 %) and heavy chain (46 %) was demonstrated to be a complex-type biantennary disialylated core-fucosylated structure. Two to 17 % of variants of this main N-glycan structure on both N-glycosylation sites had a varying degree of sialylation (0 to 1) or were incompletely processed. Another 18 % were biantennary and contained a terminal N-acetyl hexosamine (HexNAc) on one chain (galacto-type) and 0–1 terminal sialic acids on the other, or two terminal HexNAcs (4 %) corresponding to the agalacto-type. The HexNAcs were identified as GalNAcs by monosaccharide analysis (data not shown). The relative amount of agalacto and galacto-type N-glycans was higher on the heavy chain. Approximately 4 % of the N-glycans was composed of complex-type tri-antennary structures with two or three sialic acids, and some tetra-sialylated structures close to the detection limit.

Results for the N-glycan composition of CHOrFVII showed that, as for the BHKrFVII preparation, the CHOrFVII main N-glycan structure was demonstrated to be a complex-type biantennary disialylated core-fucosylated structure on the light (49 %) and heavy chain (65 %) followed by variants with differing degree of sialylation (0 to 1) and incompletely processed structures. In total, 96 % of oligosaccharides on CHOrFVII were core fucosylated, bi-antennary structures carrying no, one (35 %) or two (57 %) sialic acid residuesor incompletely processed variants. N-glycans on CHOrFVII showed lesser complexity than on the other cell lines. A similar amount of complex-type tri- and tetra-antennary, core-fucosylated structures to those of BHKrFVII was determined (4 %), as reflected in the oligosaccharide profiles (Fig. 4). The major difference in BHKrFVII and CHOrFVII N-glycan composition was the absence of GalNAcs on CHOrFVII.

Almost all N-glycans on HEK293rFVII were not comparable with those detected on or reported for pdFVII [11], or detected on rFVII from the other cell lines. Oligosaccharide mapping revealed a higher diversity and a lower amount of acidic N-glycan structures, but mostly neutral N-linked oligosaccharides (Fig. 4). About 20 different structures were detected, of which only two of those detected at higher 2 % (4 % of total structures) were observed on pdFVII (a biantennary complex-type disialylated structure, and an incomplete core-fucosylated complex-type monosialylated structure). The main N-glycan structures on HEK293rFVII were agalacto-type biantennary core-fucosylated with additional variable fucosylation (0 to 3) on the antennae. In total, 57 % of the identified structures belonged to this agalacto-type N-glycan variant, which was also most prominent on the heavy chain. Another 25 % were biantennary galacto-type core-fucosylated, with varying degrees of antennary fucosylation (0–1) and sialylation (0–2). This form was the most prominent on the light chain. The remaining 15 % were shown to be incompletely processed variants, hybrid-type structures with and without sialylation and antennary fucosylation, and high-Man structures on the light chain, which were not detected on pdFVII, or the other rFVII preparations. As for all other FVII preparations, the structural diversity on the light chain N-glycosylation site (N145) was higher. The presence of GalNAc in the HEK293rFVII preparation was revealed by monosaccharide analysis (data not shown). The tri- and tetra-antennary structures were close to detection limit. No sulfated GalNAcs were detected as reported for other HEK293-derived recombinant proteins [32].

## Discussion

The post translational modifications of rFVII derived from a human cell line e.g. the biochemical properties of rFVII produced in HEK293 cells were compared with those of rFVII expressed in CHO and BHK cell lines, and of pdFVII. Single-cell derived producer cell lines were generated, and clones selected according to growth characteristics, rFVII productivity, activity and degree of pre-activation. For the production of rFVII, cell culture and purification conditions were kept equal for all three cell lines to enable comparability of results. Although cell culture conditions were not controlled as in a bioreactor, and metabolites not measured, we assume that product qualities were comparable due to identical cell culture and purification conditions. This comparability was also reflected by highly similar degrees of γ-carboxylation in all rFVII isolates (53 % carboxylation of the 10^th^ Gla on average in CHOrFVII and BHKrFVII, and a mean ± standard deviation of 56 ± 3.7 % in four HEK293rFVII isolates). Clonal differences regarding γ-carboxylation in the purified rFVII isolates were assessed, as this parameter is known to be sensitive to limitations in the cellular post-translational protein modification machinery in the secretion pathway of recombinant vitamin K dependent coagulation factors [33]. For example, the relative standard deviation of the four HEK293rFVII isolates was 7 % for the quantification of the 10 Gla-modified peptide, and 9 % for the 9 Gla-containing peptide. We therefore concluded that our cell culture system was repeatedly able to produce rFVII from different clones and host cell lines at similarly high quality.

Consistent with the literature [10], differences were detected between the O-glycans on BHKrFVII and those on the other rFVIIa isolates. The most marked differences between rFVII from the three cell types and pdFVII were in their N-glycosylation. Unexpectedly, most N-glycans synthetized by HEK293 on rFVII were not comparable with those published for pdFVII [11], or found on our own pdFVII preparation. A high percentage of N-glycans carrying terminal Gal and GalNAcs (galacto- and agalacto-type) was detected in HEK293rFVII, a smaller degree in BHKrFVII, and only a small fraction terminating only in Gal in CHOrFVII. Data from our BHKrFVII characterization were in agreement with those reported for BHK-derived rFVIIa [34].

The N-glycans on our HEK293rFVII were similar to those described for other HEK293-derived recombinant proteins. Croset and colleagues compared the post translational modifications of recombinant proteins expressed in parallel in a CHO and a HEK293 host cell system. Proteins derived from HEK293 EBNA and HEK293-6E cell lines had similar glycostructures consisting of more heterogenous and mostly basic isoforms compared with CHO, indicating a lower amount of sialic acids [35]. Recombinant activated protein C (rAPC) derived from HEK293 cells was shown to have 50 % fewer sialic acids than pdAPC, with most N-glycans ending in partially sialylated GalNAc, and to contain 5-fold more Fuc [36]. APC is cleared via complex formation with its inhibitors [37, 38] as demonstrated by a comparably short t_1/2_ of rAPC (8–12 min) and pdAPC (12–15 min) in monkeys, and 17 min in humans [39].

Other proteins derived from HEK293 have been described: L-selectin with mainly core- and antennary-fucosylated biantennary N-glycans terminating in either Gal or GalNAc [40], and a human leukocyte receptor IIIa (FcγRIIIa). The faster association and dissociation of IgG on the CHO- than on the HEK293-derived recombinant FcγRIIIa was attributed to the observed differences in glycosylation [41].

It is currently unknown whether Fuc attached to the antennary structures impair rFVIIa function and clearance. As we did not clarify glycan linkages in detail, it was not possible to verify or exclude the presence of ABH blood group antigens on HEK293rFVII. These have been reported to influence plasma levels of circulating von Willebrand factor, and FVIII [42].

HEK293 cells are currently in use to develop recombinant protein products for hemophilia therapy, i.e. a B-domain-deleted FVIII [43], and FVIIa-Fc, FIX-Fc, and FVIII-Fc fusion proteins [44–46]. They were reported to produce interferon-α2b which was highly similar to its natural counterpart [47]. N-glycans on HEK293 derived B-domain deleted FVIII were recently characterized [48]. The main structure found on pdFVIII (~50 % of N-glycans) - a biantennary complex type terminating in Gal capped with one or two sialic acids with and without core fucosylation [49] - was detected in <10 % of all HEK293rFVIII N-linked carbohydrates. Similar structures as on our HEK293rFVII, such as biantennary N-glycans terminating in Gal and GalNAc with core and antenna fucosylation, were found on HEK293-derived rFVIII. A comparison of recombinant human erythropoietin derived from various cell lines revealed a lower degree of sialylation for the protein from HEK293 than from CHO or BHK cells [50].

Post translational modifications, particularly glycosylation patterns, influence the pharmacokinetics and pharmacodynamics of recombinant proteins. Gal and GalNAc have been described to bind the asialoglycoprotein receptor with high affinity [51], leading to rapid clearance from the circulation. We therefore assumed CHOrFVII to be the most suitable precursor for a therapeutic rFVIIa product as it had the highest degree of sialylation and no terminal GalNAc. Significantly higher areas under the curve (AUCs) for CHOrFVIIa than for BHKrFVIIa observed in healthy volunteers and hemophilia patients [16] suggest that intravenously injected HEK293rFVIIa would remain shortest in circulation, although clinical and preclinical confirmation of this assumption is lacking. In a similar study, we showed that HEK293rFIX has a significantly lower IVR and AUC than CHOrFIX in FIX knock-out mice [52]. HEK293 derived recombinant ADAMTS13 exhibited a much shorter *in vivo* half-life in mice than CHO produced rADAMTS13 [53]. In summary, several studies comparing features of recombinant proteins derived from HEK293 cells with those from other cell lines or the naturally occurring form found lower degrees of terminal sialic acids on the N-glycans, high amounts of fucosylation, glycan forms not found on the natural counterpart, and faster clearance from circulation for the HEK293-derived protein. Another concern when using a human cell line to produce a human therapeutic would be human cell derived host cell contaminations. There is a currently unknown risk that co-administration of contaminating human host-cell derived proteins could induce, over time and after repeated dosing, anti-host cell protein antibodies which might behave like human auto-antibodies and thus increase the risk of adverse reactions in patients.

## Conclusion

In conclusion, the only major differences in post-translational modifications between HEK293rFVII and rFVII expressed in CHO, BHK cells and pdFVII were in N-glycosylation, with most N-glycans on HEK293 differing from those on rFVII expressed in other two cell lines, and, surprisingly, from pdFVII. From all rFVII isolates investigated, CHOrFVII contained the highest degree of sialylation and no terminal GalNAc, with all other protein features of high quality at high productivity, and CHO cells were therefore assumed to be the best choice for the production of rFVII. These results emphasize the importance of assessing the suitability of a cell line, or a particular cell clone, for producing human therapeutic proteins on a case-by-case basis.

## Additional file

Additional file 1:
**An Additional File in PDF format (“Additional file_rFVIIthreecelltypes_ EBoehm_REVISION072015.pdf”) is provided containing the** **LC-MS** **raw data for all FVII isolates concerning** **γ-carboxylation** **(Tables S1 and S2)**, **O-glycosylation** **(Tables S3), and N-glycosylation (Table S4).** (PDF 1065 kb)

## Abbreviations

ATCC: American type culture collection
AUC: Area under the curve
BHK: Baby hamster kidney
CHO: Chinese hamster ovary
DHFR: Dihydro-folate reductase
DTT: Dithio-threitol
ELISA: Enzyme linked immune-sorbent assay
FBS: Fetal bovine serum
Fuc: Fucose
FVII: Coagulation factor VII
FVIII: Coagulation factor VIII
Gal: Galactose
GalNAc: N-acetyl galactosamine
Gla: γ-carboxy-glutamate
Glc: Glucose
GlcNAc: N-acetyl glucosamine
HEK: Human embryonic kidney
HexNAc: N-acetyl hexosamine
HPAEC-PAD: High pH anion exchange chromatography – pulse amperometric detection
HPLC: High performance liquid chromatography
IVR: *In vivo* recovery
LC-MS: Liquid chromatography followed by mass spectrometry
Man: Mannose
MTX: Methotrexate
NANA: N-acetyl neuraminic acid
NGNA: N-glycolyl neuraminic acid
PCR: Polymerase chain reaction
Pd: Plasma derived
pdAPC: Plasma-derived activated protein C
PT: Prothrombin time
rAPC: Recombinant activated protein C
rFVIIa: Recombinant activated coagulation factor VII
SDS-PAGE: Sodium-dodecylsulfate polyacrylamide gel electrophoresis
TF: Tissue factor
Xyl: Xylose
