# Supplementary material for: Differences in N-glycosylation of recombinant human coagulation factor VII derived from BHK, CHO, and HEK293 cells
Source: BMC Biotechnol. 2015 Sep 18;15:87. doi: 10.1186/s12896-015-0205-1 (PMC4574471; doi:10.1186/s12896-015-0205-1)
Supplement: Additional file 1: — An Additional File in PDF format (“Additional file_rFVIIthreecelltypes_ EBoehm_REVISION072015.pdf”) is provided containing the LC-MS raw data for all FVII isolates concerning γ-carboxylation (Tables S1 and S2), O-glycosylation (Tables S3), and N-glycosylation (Table S4). (PDF 1065 kb) [file 12896_2015_205_MOESM1_ESM.pdf]

Additional file to “Differences in N-glycosylation of recombinant human coagulation factor VII derived from BHK, CHO, and HEK293 cells”

Table S1A to D: Data and relative abundances of the γ-carboxylated peptides of FVII isolates as detected by LC-MS after tryptic digest. Detected and corresponding theoretical masses and their differences for the respective tryptic peptides containing 8, 9 or 10 Gla residues (at E6, E7, E14, E16, E19, E20, E25, E26, E29, and E35) are shown. For structures detected on more than one clone, arithmetic means of relative abundances were calculated. For HEK293rFVII (Tables S1A), for which isolates from four clones were analyzed in parallel, the standard deviations and relative standard deviations were also calculated. Tables S1B: BHKrFVII; S1C: CHOrFVII; S1D: pdFVII.

Table S1A: HEK293rFVII:

Gamma-carboxylated peptide	r_t [min]	Molecular mass [Da]	Molecular $mass_{theoret.}$ [Da]	Δ_{mass} [Da]	Intensity [arbitrary units]	Relative abundance [%]	Mean relative abundance [%]	Standard deviation [%]	Relative standard deviation [%]
T1-5 (10 Gla´s)	30.1-31.5	4816.41	4814.89	-1.52	31.2	23.9	16	7	46
	30.1-31.5	4817.00	4814.89	-2.11	24.7	7.0			
	30.1-31.5	4815.18	4814.89	-0.29	22.8	13.7			
	30.1-31.5	4816.67	4814.89	-1.78	48.3	20.3			
T1-5 (8 Gla´s)	30.1-31.5	4725.77	4726.91	1.14	7.07	5.4	6	1	8
	30.1-31.5	4726.58	4726.91	0.33	23.2	6.6			
	30.1-31.5	4725.25	4726.91	1.66	10.4	6.3			
	30.1-31.5	4727.27	4726.91	-0.36	14.5	6.1			
T1-5 (9 Gla´s)	30.1-31.5	4770.61	4770.90	0.29	92.5	70.7	78	7	9
	30.1-31.5	4770.59	4770.90	0.31	305	86.4			
	30.1-31.5	4769.77	4770.90	1.13	133	80.0			
	30.1-31.5	4771.09	4770.90	-0.19	175	73.6			
T1-6 (10 Gla´s)	29.95	5043.76	5044.17	0.41	93.7	96.2	96	0.1	0.1
	29.89	5043.87	5044.17	0,30	181	96.3			

Gamma-carboxylated peptide	r_t [min]	Molecular mass [Da]	Molecular $mass_{theoret.}$ [Da]	Δ_{mass} [Da]	Intensity [arbitrary units]	Relative abundance [%]	Mean relative abundance [%]	Standard deviation [%]	Relative standard deviation [%]
	29.91	5042.91	5044.17	1.26	168	96.2			
	29.91	5044.72	5044.17	-0.55	364	96.4			
T1-6 (9 Gla´s)	29.95	5000.01	5000.18	0.17	3.7	3.8	4	0.1	3
	29.89	4999.86	5000.18	0.32	6.9	3.7			
	29.91	4998.92	5000.18	1.26	6.7	3.8			
	29,.1	5000.38	5000.18	-0.20	13.7	3.6			

Table S1B: BHKrFVII:

Gamma-carboxylated peptide	r_t [min]	Molecular mass [Da]	Molecular $mass_{theoret.}$ [Da]	Δ_{mass} [Da]	Intensity [arbitrary units]	Relative abundance [%]
T1-5 (10 Gla´s)	30.1-31,5	4816.63	4814.89	-1.74	40.6	9.5
T1-5 (9 Gla´s)	30.1-31,5	4770.56	4770.90	0.34	367	85.8
T1-5 (8 Gla´s)	30.1-31,5	4726.67	4726.91	0.24	20.1	4.7
T1-6 (10 Gla´s)	29.95	5044.25	5044.17	-0.08	90.0	96.1
T1-6 (9 Gla´s)	29.95	4999.66	5000.18	0.52	3.7	3.9

Table S1C: CHOrFVII:

Gamma-carboxylated peptide	r_t [min]	Molecular mass [Da]	Molecular $mass_{theoret.}$ [Da]	Δ_{mass} [Da]	Intensity [arbitrary units]	Relative abundance [%]	Mean relative abundance [%]
T1-5 (10xGla)	30.1-31.5	4816.17	4814.89	-1.28	13.1	10.7	9.9
	30.1-31.5	4815.39	4814.89	-0.50	3.13	9.1	
T1-5 (8xGla)	30.1-31.5	4726.34	4726.91	0.57	11.8	9.6	9.7
	30.1-31.5	4727.57	4726.91	-0.66	3.35	9.7	
T1-5 (9xGla)	30.1-31.5	4770.31	4770.90	0.59	97.8	79.7	80.5
	30.1-31.5	4771.10	4770.90	-0.20	28.1	81.3	
T1-6 (10xGla)	29.95	5043.55	5044.17	0.62	63.7	96.4	95.5
	29.74	5044.49	5044.17	-0.32	20.5	94.5	
T1-6 (9xGla)	29.95	5000.72	5000.18	-0.54	2.38	3.6	4.6
	29.74	4999.95	5000.18	0.23	1.19	5.5	

Table S1D: pdFVII:

Gamma-carboxylated peptide	r_t [min]	Molecular mass [Da]	Molecular $mass_{theoret.}$ [Da]	Δ_{mass} [Da]	Intensity [arbitrary units]	Relative abundance [%]
T1-5 (10xGla)	30.1-31.2	4814.20	4814.89	0.69	343	67.3
T1-5 (8xGla)	30.1-31.2	4724.98	4726.91	1.93	41	8
T1-5 (9xGla)	30.1-31.2	4770.35	4770.90	0.55	126	24.7
T1-6 (10xGla)	29.71	5043.74	5044.17	0.43	3060	96.3
T1-6 (9xGla)	29.71	4999.73	5000.18	0.45	117	3.7

Table S2: Comparison of γ-carboxylation between clones and host cell lines.

Relative percentages of peptides with 9 and 10 Gla residues for CHOrFVII isolates from two clones, BHKrFVII isolates from one clone, and HEK293rFVII isolates from four clones are shown. Arithmetic means were calculated for CHOrFVII and HEK293rFVII; for HEK293rFVII, standard deviations between clones were also calculated.

**CHOrFVII**			**BHKrFVII**			**HEK293rFVII**		
Clone 1	10 Gla´s	54%	Clone 1	10 Gla´s	53%	Clone 1	10 Gla´s	60%
	9 Gla´s	42%		9 Gla´s	45%		9 Gla´s	37%
Clone 2	10 Gla´s	52%				Clone 2	10 Gla´s	52%
	9 Gla´s	43%					9 Gla´s	45%
**Mean**	**10 Gla´s**	**53%**				Clone 3	10 Gla´s	55%
	**9 Gla´s**	**43%**					9 Gla´s	42%
						Clone 4	10 Gla´s	58%
							9 Gla´s	39%
						**Mean ± standard deviation**	**10 Gla´s**	**56 ± 3.7%**
							**9 Gla´s**	**41 ± 3.5%**

Table S3A-D: Data and relative abundances of the O-glycosylated peptides of rFVII isolates as detected by LC-MS after tryptic digest. Detected and corresponding theoretical masses and their differences for the respective tryptic peptides

modified with the proposed O-glycan stuctures on S52 and S60 are shown. For structures detected on more than one clone, arithmetic means of relative abundances were calculated. For HEK293rFVII (Table S3A), for which isolates from four clones were analyzed in parallel, the standard deviations and relative standard deviations were also calculated. Tables S3B: BHKrFVII; S3C: CHOrFVII; S3D: pdFVII. Abbreviations: Fuc: fucose; Glc: glucose; Xyl: xylose.

Table S3A: HEK293rFVII

Peptide T7 detected	r_t [min]	Molecular mass [Da]	Molecular $mass_{theoret.}$ [Da]	Δ_{mass} [Da]	Intensity [arbitrary units]	Relative abundance [%]	Mean relative abundance [%]	Standard deviation [%]	Relative standard deviation [%]
T7	24.9-25.5	2724.22	2723.10	-1.12	31.3	3.8	3.7	0.3	8
	24.9-25.5	2724,29	2723.10	-1.19	33,2	4.1			
	24.9-25.5	2723.80	2723.10	-0.70	20,9	3.5			
	24.9-25.5	2724.20	2723.10	-1.10	35,3	3.5			
T7 Fuc	24.9-25.5	2870.13	2869.24	-0.89	26,2	3.2	3.4	0.4	11
	24.9-25.5	2870.18	2869.24	-0.94	29.6	3.6			
	24.9-25.5	2869.64	2869.24	-0.40	22.2	3.8			
	24.9-25.5	2870.23	2869.24	-0.99	30.0	3			
T7 Fuc Glc	24.9-25.5	3031.46	3031.39	-0.07	101	12.3	10.0	4.4	44
	24.9-25.5	3031.48	3031.39	-0.09	121	14.9			
	24.9-25.5	3030.49	3031.39	0.90	39.0	6.6			
	24.9-25.5	3031.84	3031.39	-0.45	59.8	6			
T7 Fuc GlcXyl	24.9-25.5	3164.49	3163.50	-0.99	61.2	7.4	7.2	0.2	3
	24.9-25.5	3164.39	3163.50	-0.89	55.9	6.9			
	24.9-25.5	3163.63	3163.50	-0.13	43.6	7.4			
	24.9-25.5	3164.80	3163.50	-1.30	71.1	7.1			
T7 Fuc $GlcXyl_2$	24.9-25.5	3296,.00	3295.62	-0.38	487	59.2	61.8	4.9	8
	24.9-25.5	3296.63	3295.62	-1.01	460	56.6			
	24.9-25.5	3295.69	3295.62	-0.07	373	63.3			
	24.9-25.5	3296.89	3295.62	-1.27	682	67.9			
T7 Glc	24.9-25.5	2885.99	2885.24	-0.75	43.1	5.2	4.6	1.1	25

Peptide T7 detected	r_t [min]	Molecular mass [Da]	Molecular $mass_{theoret.}$ [Da]	Δ_{mass} [Da]	Intensity [arbitrary units]	Relative abundance [%]	Mean relative abundance [%]	Standard deviation [%]	Relative standard deviation [%]
	24.9-25.5	2886.01	2885.24	-0.77	46.6	5.7			
	24.9-25.5	2885.68	2885.24	-0.44	25.6	4.3			
	24.9-25.5	2886.64	2885.24	-1.40	31.2	3.1			
T7 GlcXyl	24.9-25.5	3018.29	3017.36	-0.93	21.4	2.6	2.7	0.6	22
	24.9-25.5	3018.35	3017.36	-0.99	18.6	2.3			
	24.9-25.5	3017.83	3017.36	-0.47	21.0	3.6			
	24.9-25.5	3018.62	3017.36	-1.26	24.5	2.4			
T7 $GlcXyl_2$	24.9-25.5	3150.35	3149.47	-0.88	51.8	6.3	6.7	0.6	10
	24.9-25.5	3150.44	3149.47	-0.97	48.5	6			
	24.9-25.5	3149.41	3149.47	0.06	43.8	7.4			
	24.9-25.5	3150.77	3149.47	-1.30	70.8	7			

Table S3B: BHKrFVII

Peptide T7 detected	r_t [min]	Molecular mass [Da]	Molecular $mass_{theoret.}$ [Da]	Δ_{mass} [Da]	Intensity [arbitrary units]	Relative abundance [%]
T7	24.9-25.5	2723.00	2723.10	0.10	82.4	5
T7 Fuc	24.9-25.5	2870.27	2869.24	-1.03	78.0	4.7
T7 Fuc Glc	24.9-25.5	3032.48	3031.39	-1.09	830	50.4
T7 Fuc GlcXyl	24.9-25.5	3164.63	3163.50	-1.13	46.0	2.8
T7 Fuc $GlcXyl_2$	24.9-25.5	3296.49	3295.62	-0.87	343	20.8
T7 Glc	24.9-25.5	2886.41	2885.24	-1.17	199	12.1
T7 GlcXyl	24.9-25.5	3017.11	3017.36	0.25	21.7	1.3
T7 $GlcXyl_2$	24.9-25.5	3150.44	3149.47	-0.97	46.7	2.8

Table S3C: CHOrFVII

Peptide T7 detected	r_t [min]	Molecular mass [Da]	Molecular $mass_{theoret.}$ [Da]	Δ_{mass} [Da]	Intensity [arbitrary units]	Relative abundance [%]	Mean relative abundance [%]	Standard deviation [%]
T7	24.9-25.5	2723.79	2723.10	-0.69	15.8	3.7	3.9	0.3

Peptide T7 detected	r_t [min]	Molecular mass [Da]	Molecular mass$_{theoret.}$ [Da]	Δ_{mass} [Da]	Intensity [arbitrary units]	Relative abundance [%]	Mean relative abundance [%]	Standard deviation [%]
	24.7-25.3	2723.91	2723.10	-0.81	8.20	4.1		
T7 Fuc	24.9-25.5	2869.85	2869.24	-0.61	14.5	3.4	3.5	0.1
	24.7-25.3	2870.76	2869.24	-1.52	7.28	3.6		
T7 Fuc Glc	24.9-25.5	3030.79	3031.39	0.60	50.9	12	13.8	2.6
	24.7-25.3	3031.30	3031.39	0.09	31.3	15.6		
T7 Fuc GlcXyl	24.9-25.5	3164.10	3163.50	-0.60	27.4	6.4	6.8	0.6
	24.7-25.3	3164.23	3163.50	-0.73	14.4	7.2		
T7 Fuc GlcXyl$_2$	24.9-25.5	3296.13	3295.62	-0.51	251	59	56.1	4.1
	24.7-25.3	3296.68	3295.62	-1.06	107	53.2		
T7 Glc	24.9-25.5	2886.05	2885.24	-0.81	20.1	4.7	6	1.8
	24.7-25.3	2886.32	2885.24	-1.08	14.7	7.3		
T7 GlcXyl	24.9-25.5	3018.24	3017.36	-0.88	12.8	3	3	0.0
	24.7-25.3	3018.79	3017.36	-1.43	5.96	3		
T7 GlcXyl$_2$	24.9-25.5	3149.79	3149.47	-0.32	32.7	7.7	6.9	1.1
	24.7-25.3	3150.44	3149.47	-0.97	12.3	6.1		

Table S3D: pdFVII

Peptide T7 detected	r_t [min]	Molecular mass [Da]	Molecular mass$_{theoret.}$ [Da]	Δ_{mass} [Da]	Intensity [arbitrary units]	Relative abundance [%]
T7	24.83	2724.10	2723.10	-1.0	99.6	5.6
T7 Fuc	24.83	2869.67	2869.24	-0.43	66.6	3.8
T7 Fuc Glc	24.83	3030.68	3031.39	0.71	26.2	14.8
T7 Fuc GlcXyl	24.83	3164.31	3163.50	-0.81	477	27.0
T7 Fuc GlcXyl$_2$	24.83	3296.37	3295.62	-0.75	545	30.8
T7 Glc	24.83	2886.15	2885.24	-0.91	131	7.4
T7 GlcXyl	24.83	3018.17	3017.36	-0.81	112	6.3
T7 GlcXyl$_2$	24.83	3150.22	3149.47	-0.75	74.8	4.2

Table S4A-H: Data and relative abundances for N-glycosylated peptides of CHOrFVII, HEK293rFVII, BHKrFVII clones and pdFVII from the corresponding light and heavy chains, as detected by LC-MS. Detected and corresponding theoretical masses and their differences for the respective tryptic peptides modified with the proposed N-glycan stuctures are shown. For structures detected on more than one clone, arithmetic means of relative abundances were calculated. For HEK293rFVII (Tables S4A, light chain, and S4B, heavy chain), for which isolates from four clones were analyzed in parallel, the standard deviations and relative standard deviations were also calculated. Tables S4C and S4D: BHKrFVII; S4E and S4F: CHOrFVII; S4G and S4H: pdFVII. Abbreviations: A1: one antenna completed with GlcNAc and Gal on the oligosaccharide core. A2: two antennae completed with GlcNac and Gal on the oligosaccharide core. A3: three antennae completed with GlcNac and Gal on the oligosaccharide core. Man: high mannose structure. S: sialic acid. HexNAc: N-acetyl hexosamine (GlcNAc or GalNAc); F: fucose. Hex: hexose (Gal or Man). Txx: tryptic peptide plus identifier. For example, A1FS1+HexNAc2 is a mono-fucosylated biantennary structure with one antenna completed with GlcNAc and Gal and one sialic acid, and another consisting of two HexNAcs. HexNAc4F3 is a biantennary structure consisting of two HexNAcs on each antenna, and containing three fucoses. A3FS2 is a triantennary structure containing two sialic acids and one fucose.

Table S4A: HEK293rFVII N145 (light chain):

N-glycosylated peptide	r_t [min]	Molecular mass [Da]	Molecular $mass_{theoret.}$ [Da]	Δ_{mass} [Da]	Intensity [arbitrary units]	Relative abundance [%]	Mean relative abundance [%]	Standard deviation [%]	Relative standard deviation [%]
T16 A2F+Hex	5.5-7.0	2787.35	2787.14	-0.21	11.2	5.2	3.1	1.7	56
	5.5-7.0	2787.28	2787.14	-0.14	9.9	3.5			
	5.5-7.0	2786.53	2787.14	0.61	4.2	2.3			
	5.5-7.0	2786.68	2787.14	0.46	4.1	1.2			
T16 A2F2+Hex	5.5-7.0	2933.68	2933.2	-0.48	7.0	3.3	3.0	0.8	26
	5.5-7.0	2933.28	2933.2	-0.08	9.6	3.4			

N-glycosylated peptide	r_t [min]	Molecular mass [Da]	Molecular $mass_{theoret.}$ [Da]	Δ_{mass} [Da]	Intensity [arbitrary units]	Relative abundance [%]	Mean relative abundance [%]	Standard deviation [%]	Relative standard deviation [%]
	5.5-7.0	2933.14	2933.2	0.06	6.2	3.4			
	5.5-7.0	2933.07	2933.2	0.13	6.4	1.8			
T16 Man6F	5.5-7.0	2866.97	2867.14	0.17	4.4	2.0	1.5	0.5	34
	5.5-7.0	2867.23	2867.14	-0.09	5.0	1.8			
	5.5-7.0	2866.52	2867.14	0.62	2.8	1.5			
	5.5-7.0	2867.01	2867.14	0.13	3.0	0.8			
T16 HexNAc2F	5.5-7.0	2300.84	2300.98	0.14	8.8	4.1	2.9	0.8	28
	5.5-7.0	2300.53	2300.98	0.45	7.7	2.7			
	5.5-7.0	2300.95	2300.98	0.03	4.2	2.3			
	5.5-7.0	2300.67	2300.98	0.31	9.0	2.5			
T16 HexNAc2F2	5.5-7.0	2447.25	2447.04	-0.21	4.2	2.0	1.9	0.1	4
	5.5-7.0	2447.01	2447.04	0.03	5.2	1.9			
	5.5-7.0	2447.07	2447.04	-0.03	3.5	1.9			
	5.5-7.0	2447.48	2447.04	-0.44	6.6	1.8			
T16 HexNAc4F2	5.5-7.0	2852.92	2853.2	0.28	46.7	21.9	17.5	3.7	21
	5.5-7.0	2853.19	2853.2	0.01	42.1	14.9			
	5.5-7.0	2853.35	2853.2	-0.15	25.6	14.0			
	5.5-7.0	2853.31	2853.2	-0.11	68.5	19.3			
T16 HexNAc4F3	5.5-7.0	2998.54	2999.26	0.72	22.6	10.6	9.7	1.6	17
	5.5-7.0	2998.74	2999.26	0.52	24.9	8.8			
	5.5-7.0	2998.27	2999.26	0.99	14.4	7.9			
	5.5-7.0	2998.85	2999.26	0.41	40.4	11.4			
T16 A1F	5.5-7.0	2259.94	2259.96	0.02	2.3	1.1	1.4	0.2	15
	5.5-7.0	2260.09	2259.96	-0.13	4.3	1.5			
	5.5-7.0	2260.1	2259.96	-0.14	3.0	1.6			
	5.5-7.0	2260.27	2259.96	-0.31	5.0	1.4			
T16 A1F+Hex3	5.5-7.0	2746.15	2746.12	-0.03	3.4	1.6	1.9	0.9	50
	5.5-7.0	2745.64	2746.12	0.48	8.2	2.9			
	5.5-7.0	2745.69	2746.12	0.43	4.3	2.3			
	5.5-7.0	2745.82	2746.12	0.30	2.5	0.7			
T16 A1F2+Hex3	5.5-7.0	2892.22	2892.17	-0.05	1.9	0.9	1.4	0.6	46

N-glycosylated peptide	r_t [min]	Molecular mass [Da]	Molecular $mass_{theoret.}$ [Da]	Δ_{mass} [Da]	Intensity [arbitrary units]	Relative abundance [%]	Mean relative abundance [%]	Standard deviation [%]	Relative standard deviation [%]
	5.5-7.0	2892.07	2892.17	0.10	6.0	2.1			
	5.5-7.0	2892.2	2892.17	-0.03	3.2	1.7			
	5.5-7.0	2891.63	2892.17	0.54	2.8	0.8			
T16 A1FS1+Hex3	5.5-7.0	3037.41	3037.21	-0.20	8.2	3.8	3.9	1.6	41
	5.5-7.0	3037.68	3037.21	-0.47	14.8	5.2			
	5.5-7.0	3037.23	3037.21	-0.02	9.2	5.0			
	5.5-7.0	3036.65	3037.21	0.56	6.2	1.7			
T16 A1F+HexNAc2	5.5-7.0	2665.8	2666.12	0.32	10.1	4.7	4.2	0.6	13
	5.5-7.0	2665.78	2666.12	0.34	12.0	4.3			
	5.5-7.0	2665.84	2666.12	0.28	8.0	4.4			
	5.5-7.0	2666.01	2666.12	0.11	12.0	3.4			
T16 A1F2+HexNAc2	5.5-7.0	2812.42	2812.17	-0.25	22.4	10.5	15.6	4.7	30
	5.5-7.0	2812.64	2812.17	-0.47	40.4	14.3			
	5.5-7.0	2812.21	2812.17	-0.04	39.8	21.8			
	5.5-7.0	2812.03	2812.17	0.14	56.4	15.9			
T16 A1F+HexNAc	5.5-7.0	2463.29	2463.04	-0.25	2.6	1.2	1.0	0.3	27
	5.5-7.0	2463.41	2463.04	-0.37	3.4	1.2			
	5.5-7.0	2463.32	2463.04	-0.28	1.4	0.8			
	5.5-7.0	2463.21	2463.04	-0.17	2.3	0.7			
T16 A1FS2+HexNAc2	5.5-7.0	3248.48	3248.31	-0.17	13.1	6.1	4.5	1.3	28
	5.5-7.0	3248.46	3248.31	-0.15	13.6	4.8			
	5.5-7.0	3248.66	3248.31	-0.35	6.0	3.3			
	5.5-7.0	3248.84	3248.31	-0.53	13.3	3.7			
T16 A1FS1	5.5-7.0	2551.26	2551.05	-0.21	3.6	1.7	1.9	0.4	19
	5.5-7.0	2551.16	2551.05	-0.11	4.4	1.6			
	5.5-7.0	2550.94	2551.05	0.11	3.4	1.9			
	5.5-7.0	2550.94	2551.05	0.11	8.7	2.4			
T16 A1FS1+HexNAc2	5.5-7.0	2957.45	2957.21	-0.24	21.4	10.0	10.2	1.8	18
	5.5-7.0	2957.5	2957.21	-0.29	34.9	12.4			
	5.5-7.0	2957.23	2957.21	-0.02	18.6	10.2			
	5.5-7.0	2957.21	2957.21	0.00	28.3	8.0			

N-glycosylated peptide	r_t [min]	Molecular mass [Da]	Molecular $mass_{theoret.}$ [Da]	Δ_{mass} [Da]	Intensity [arbitrary units]	Relative abundance [%]	Mean relative abundance [%]	Standard deviation [%]	Relative standard deviation [%]
T16 A1F2S1+HexNAc2	5.5-7.0	3103.14	3103.27	0.13	19.9	9.3	14.5	5.6	38
	5.5-7.0	3103.52	3103.27	-0.25	35.8	12.7			
	5.5-7.0	3103.52	3103.27	-0.25	25.1	13.7			
	5.5-7.0	3103.77	3103.27	-0.50	79.5	22.4			

Table S4B: HEK293rFVII N322 (heavy chain):

N-glycosylated peptide	r_t [min]	Molecular mass [Da]	Molecular $mass_{theoret.}$ [Da]	Δ_{mass} [Da]	Intensity [arbitrary units]	Relative abundance [%]	Mean relative abundance [%]	Standard deviation [%]	Relative standard deviation [%]
T31-32 HexNAc4Fuc	20.5-24.9	4277.82	4278.41	0.59	18.2	7.5	7.3	1.5	20
	20.5-24.9	4277.91	4278.41	0.50	12.5	7.2			
	20.5-24.9	4277.19	4278.41	1.22	11.8	9.1			
	20.5-24.9	4278.44	4278.41	-0.03	17.6	5.5			
T31-32 HexNAc4Fuc2	20.5-24.9	4424.23	4424.55	0.32	47.8	19.8	17.9	2.2	12
	20.5-24.9	4423.82	4424.55	0.73	25.6	14.8			
	20.5-24.9	4423.43	4424.55	1.12	23.5	18.1			
	20.5-24.9	4424.49	4424.55	0.06	60.9	19			
T31-32 HexNAc4F3	20.5-24.9	4569.94	4570.70	0.76	47.3	19.6	16.5	3.0	18
	20.5-24.9	4570.11	4570.70	0.59	24.8	14.3			
	20.5-24.9	4569.80	4570.70	0.90	17.6	13.5			
	20.5-24.9	4570.60	4570.70	0.10	59.6	18.6			
T31-32 HexNAc4F4	20.5-24.9	4715.08	4716.84	1.76	13.7	5.7	5.3	0.7	12
	20.5-24.9	4714.63	4716.84	2.21	10.4	6			
	20.5-24.9	4714.19	4716.84	2.65	6.31	4.9			
	20.5-24.9	4715.28	4716.84	1.56	14.9	4.6			
T31-33 HexNAc4F2	20.5-24.9	4914.34	4915.09	0.75	27.1	11.2	14.0	2.2	16
	20.5-24.9	4915.06	4915.09	0.03	22.8	13.2			
	20.5-24.9	4914.98	4915.09	0.11	21.1	16.2			
	20.5-24.9	4915.81	4915.09	-0.72	49.2	15.3			
T31-33 HexNAc4F3	20.5-24.9	5060.32	5061.24	0.92	22.3	9.2	12.3	3.3	27
	20.5-24.9	5060.61	5061.24	0.63	20.7	11.9			
	20.5-24.9	5059.90	5061.24	1.34	14.4	11.1			

N-glycosylated peptide	r_t [min]	Molecular mass [Da]	Molecular $mass_{theoret.}$ [Da]	Δ_{mass} [Da]	Intensity [arbitrary units]	Relative abundance [%]	Mean relative abundance [%]	Standard deviation [%]	Relative standard deviation [%]
	20.5-24.9	5061.00	5061.24	0.24	54.7	17			
T32 A2S2	20.5-24.9	4504.53	4504.50	-0.03	11.6	4.8	5.2	1.3	26
	20.5-24.9	4504.68	4504.50	-0.18	10.8	6.2			
	20.5-24.9	4503.66	4504.50	0.84	8.04	6.2			
	20.5-24.9	4504.87	4504.50	-0.37	10.8	3.4			
T32 HexNAc4F3	20.5-24.9	4440.54	4442.52	1.98	23.5	9.7	8.3	1.2	15
	20.5-24.9	4441.55	4442.52	0.97	15.6	9			
	20.5-24.9	4439.42	4442.52	3.10	9.31	7.2			
	20.5-24.9	4441.91	4442.52	0.61	23.8	7.4			
T32 A1FS1+HexNAc	20.5-24.9	4196.93	4197.24	0.31	9.4	3.9	5.0	2.5	50
	20.5-24.9	4197.80	4197.24	-0.56	14.4	8.3			
	20.5-24.9	4197.78	4197.24	-0.54	6.9	5.3			
	20.5-24.9	4197.92	4197.24	-0.68	8.0	2.5			
T32-33 HexNAc3F3	20.5-24.9	4729.15	4729.86	0.71	10.6	4.4	3.8	1.1	28
	20.5-24.9	4728.52	4729.86	1.34	8.2	4.7			
	20.5-24.9	4727.97	4729.86	1.89	4.9	3.8			
	20.5-24.9	4729.43	4729.86	0.43	7.5	2.3			
T32-33 HexNAc4F2	20.5-24.9	4786.87	4786.91	0.04	9.8	4.1	4.4	0.3	6
	20.5-24.9	4787.11	4786.91	-0.20	7.5	4.3			
	20.5-24.9	4786.15	4786.91	0.76	6.1	4.7			
	20.5-24.9	4787.20	4786.91	-0.29	13.9	4.3			

Table S4C: BHKrFVII N145 (light chain)

N-glycosylated peptide	r_t [min]	Molecular mass [Da]	Molecular $mass_{theoret.}$ [Da]	Δ_{mass} [Da]	Intensity [arbitrary units]	Relative abundance [%]
T16 A2F	5.5-7.0	2624.9	2625.09	0.19	17.4	2.0
T16 A2FS2	5.5-7.0	3207.08	3207.28	0.2	485	54.8
T16 A2FS1	5.5-7.0	2916.49	2916.16	-0.33	146	16.5

N-glycosylated peptide	r_t [min]	Molecular mass [Da]	Molecular mass$_{theoret.}$ [Da]	Δ_{mass} [Da]	Intensity [arbitrary units]	Relative abundance [%]
T16 HexNAc2Fuc	5.5-7.0	2300.63	2300.98	0.35	5.6	0.6
T16 A1F	5.5-7.0	2260.07	2259.96	-0.11	7.0	0.8
T16 A1F+HexNAc	5.5-7.0	2463.06	2463.04	-0.02	7.5	0.8
T16 A1FS1	5.5-7.0	2551.18	2551.05	-0.13	51.6	5.8
T16 A1FS1+HexNAc2	5.5-7.0	2956.99	2957.21	0.22	53.2	6.0
T16 A1FS1+HexNAc1	5.5-7.0	2754.45	2754.13	-0.32	35.9	4.1
T16 A3FS2	5.5-7.0	3572.86	3572.41	-0.45	29.3	3.3
T16 A3FS3	5.5-7.0	3862.83	3863.51	0.68	32.9	3.7

Table S4D: BHKrFVII N322 (heavy chain)

N-glycosylated peptide	r_t [min]	Molecular mass [Da]	Molecular mass$_{theoret.}$ [Da]	Δ_{mass} [Da]	Intensity [arbitrary units]	Relative abundance [%]
T31-32 A2FS2	20.5-24.9	4778.45	4778.82	0.37	257	28.2
T31-32 A2FS1	20.5-24.9	4487.26	4487.56	0.30	42.7	4.7
T31-32 HexNAc4Fuc	20.5-24.9	4278.68	4278.41	-0.27	63.7	7
T31-32 A1F+HexNAc2	20.5-24.9	4237.21	4237.36	0.15	23.7	2.6
T31-32 A1FS1+HexNAc2	20.5-24.9	4528.04	4528.62	0.58	130	14.2
T31-32 A1FS1+HexNAc1	20.5-24.9	4325.92	4325.42	-0.50	78.2	8.6
T31-32 A3FS3	20.5-24.9	5435.06	5435.42	0.36	30.3	3.3
T31-33 A3FS2	20.5-24.9	5269.48	5269.36	-0.12	79.1	8.7
T31-33 A1FS1+HexNAc2	20.5-24.9	5019.06	5019.16	0.10	59.9	6.6
T32 A2FS2	20.5-24.9	4650.56	4650.64	0.08	83.0	9.1
T32 A1FS1+HexNAc2	20.5-24.9	4400.16	4400.44	0.28	50.9	5.6
T32 A1FS1+HexNAc1	20.5-24.9	4197.15	4197.24	0.09	14.0	1.5

Tables S4E: CHOrFVII N145 (light chain)

N-glycosylated peptide	r_t [min]	Molecular mass [Da]	Molecular mass$_{theoret.}$ [Da]	Δ_{mass} [Da]	Intensity [arbitrary units]	Relative abundance [%]	Mean relative abundance [%]
T16 A2F	5.5-7.0	2624.80	2625.09	0.29	7.8	6.0	7.0
	5.3-6.9	2625.05	2625.09	0.04	4.64	7.9	
T16 A2FS2	5.5-7.0	3207.01	3207.28	0.27	61.8	47.8	48.8

N-glycosylated peptide	r_t [min]	Molecular mass [Da]	Molecular $mass_{theoret.}$ [Da]	Δ_{mass} [Da]	Intensity [arbitrary units]	Relative abundance [%]	Mean relative abundance [%]
	5.3-6.9	3207.27	3207.28	0.01	29.2	49.7	
T16 A2FS1	5.5-7.0	2916.29	2916.16	-0.13	34.2	26.5	26.7
	5.3-6.9	2916.27	2916.16	-0.11	15.8	26.9	
T16 A1F	5.5-7.0	2260.44	2259.96	-0.48	2.72	2.1	not applicable
T16 A1F+HexNAc1	5.5-7.0	2463.45	2463.04	-0.41	2.2	1.7	not applicable
T16 A1FS1	5.5-7.0	2550.97	2551.05	0.08	6.68	5.2	5.8
	5.3-6.9	2550.98	2551.05	0.07	3.68	6.3	
T16 A1FS1+HexNAc1	5.5-7.0	2752.82	2754.13	1.31	3.44	2.7	not applicable
T16 A3FS2	5.5-7.0	3572.93	3572.41	-0.52	4.08	3.2	4.3
	5.3-6.9	3572.06	3572.41	0.35	3.12	5.3	
T16 A3FS3	5.5-7.0	3863.51	3863.51	0.00	3.16	2.4	3.2
	5.3-6.9	3863.27	3863.51	0.24	2.28	3.9	

Table S4F: CHOrFVII N322 (heavy chain)

N-glycosylated peptide	r_t [min]	Molecular mass [Da]	Molecular $mass_{theoret.}$ [Da]	Δ_{mass} [Da]	Intensity [arbitrary units]	Relative abundance [%]	Mean relative abundance [%]
T31-32 A2FS2	20.5-24.9	4778.06	4778.82	0.76	37.9	37.0	37.4
	20.5-24.9	4778.52	4778.82	0.30	17.8	37.7	
T31-32 A2FS1	20.5-24.9	4487.55	4487.56	0.01	15.1	14.7	14.1
	20.5-24.9	4488.32	4487.56	-0.76	6.36	13.5	
T31-33 A2FS2	20.5-24.9	5268.46	5269.36	0.90	19.7	19.2	20.7
	20.5-24.9	5269.36	5269.36	0.00	10.5	22.2	
T31-33 A2FS1	20.5-24.9	4978.06	4978.10	0.04	13.8	13.5	14.0
	20.5-24.9	4978.40	4978.10	-0.30	6.86	14.5	
T32 A2FS2	20.5-24.9	4650.70	4650.64	-0.06	8.87	8.7	7.1
	20.5-24.9	4650.86	4650.64	-0.22	2.53	5.4	
T32 A1FS1+HexNAc	20.5-24.9	4195.55	4197.24	1.69	7.12	6.9	6.8
	20.5-24.9	4195.87	4197.24	1.37	3.17	6.7	

Table S4G: pdFVII N145 (light chain)

N-glycosylated peptide	r_t [min]	Molecular mass [Da]	Molecular $mass_{theoret.}$ [Da]	Δ_{mass} [Da]	Intensity [arbitrary units]	Relative abundance [%]
T16 A2S1	5.3-6.7	2770.30	2770.13	-0.17	59.3	7.3
T16 A2S2	5.3-6.7	3061.22	3061.10	-0.12	286	35.3
T16 A2FS2	5.3-6.7	3207.19	3207.28	0.09	56.0	6.9
T16 A2FS1	5.3-6.7	2916.22	2916.16	-0.06	15.2	1.9
T16 A1	5.3-6.7	2113.98	2113.90	-0.08	9.64	1.2
T16 A1S1	5.3-6.7	2405.36	2404.99	-0.37	78.3	9.7
T16 A1FS1	5.3-6.7	2551.40	2551.05	-0.35	12.2	1.5
T16 A1S1+HexNAc	5.3-6.7	2608.27	2608.07	-0.20	6.36	0.8
T16 A3S2	5.3-6.7	3426.91	3426.35	-0.56	69.2	8.5
T16 A3FS2	5.3-6.7	3572.93	3572.41	-0.52	33.9	4.2
T16 A3S3	5.3-6.7	3718.00	3717.45	-0.55	101	12.5
T16 A3FS3	5.3-6.7	3863.42	3863.51	0.09	54.0	6.7

Table S4H: pdFVII N322 (heavy chain)

N-glycosylated peptide	r_t [min]	Molecular mass [Da]	Molecular $mass_{theoret.}$ [Da]	Δ_{mass} [Da]	Intensity [arbitrary units]	Relative abundance [%]
T31-32 A2S2	21-25	4632.00	4632.68	0.68	535	50.1
T31-32 A2FS2	21-25	4778.00	4778.82	0.82	98.4	9.2
T31-32 A3S3	21-25	5288.87	5289.27	0.40	56.2	5.3
T31-32 A3FS3	21-25	5434.50	5435.42	0.92	26,2	2.5
T31-33 A2S2	21-25	5122.75	5123.22	0.47	222	20.8
T31-33 A2FS2	21-25	5268.28	5269.36	1.08	34.8	3.3
T31-33 A3S3	21-25	5779.36	5779.81	0.45	22.0	2.1
T31-33 A3FS3	21-25	5925.82	5925.96	0.14	11.4	1.1
T32-33 A2S2	21-25	4994.78	4995.04	0.26	62.3	5.8
